# Invasive Cancer Incidence and Survival — United States, 2011

**Published:** 2015-03-13

**Authors:** S. Jane Henley, Simple D. Singh, Jessica King, Reda Wilson, Mary Elizabeth O’Neil, A. Blythe Ryerson

**Affiliations:** 1 Division of Cancer Prevention and Control, National Center for Chronic Disease Prevention and Health Promotion, CDC

Because of improvements in early detection and treatment of cancer, the proportion of persons with cancer who survive ≥5 years after diagnosis has increased (1). To assess progress toward achieving *Healthy People 2020* objectives (2),* CDC analyzed data from U.S. Cancer Statistics (USCS) for 2011, the most recent data available. USCS includes incidence and survival data from CDC’s National Program of Cancer Registries (NPCR) and the National Cancer Institute’s Surveillance, Epidemiology, and End Results (SEER) program and mortality data from the National Vital Statistics System (3). In 2011, a total of 1,532,066 invasive cancers were reported to cancer registries in the United States (excluding Nevada), for an annual incidence rate of 451 cases per 100,000 persons. Cancer incidence rates were higher among males (508) than females (410), highest among black persons (458), and ranged by state, from 374 to 509 per 100,000 persons (339 in Puerto Rico). The proportion of persons with cancer who survived ≥5 years after diagnosis was 65% and was similar among males (65%) and females (65%) but lower among black persons (60%) compared with white persons (65%). Surveillance of cancer incidence and survival are essential for identifying population groups with high cancer incidence rates and low cancer survival rates as well as for estimating the number of cancer survivors, which was 13.7 million in 2012 (1). These data are being used by states to effectively develop comprehensive cancer control programs, including supporting the needs of cancer survivors.

Invasive cancers are all cancers excluding in situ cancers (except in the urinary bladder) and basal and squamous cell skin cancers. Data on new cases of invasive cancer diagnosed during 2011 were obtained from population-based cancer registries affiliated with the NPCR and/or SEER programs in each state, the District of Columbia (DC), and Puerto Rico (3). For comparability with past estimates, data for the United States are restricted to the states and DC, and data for Puerto Rico are analyzed and presented separately. Data from DC and all states except Nevada met USCS publication criteria for 2011†; consequently, data in this report cover 99% of the U.S. population. Cases were first classified by anatomic site using the *International Classification of Diseases for Oncology, Third Edition*. Cases with hematopoietic histologies were further classified using the *World Health Organization Classification of Tumours of Haematopoietic and Lymphoid Tissues, Fourth Edition*. Breast cancers were characterized by stage at diagnosis using SEER *Summary Staging Manual 2000*§; late-stage cancers include those diagnosed after they had spread regionally or metastasized.

Population denominators for incidence rates are race-, ethnicity-, and sex-specific county population estimates from the 2010 U.S. Census, as modified by SEER and aggregated to the state and national level.¶ Annual incidence rates per 100,000 population were age-adjusted by the direct method to the 2000 U.S. standard population.

For the first time, a subset of the USCS dataset includes the 5-year relative survival rate, defined as the proportion of persons surviving ≥5 years after cancer diagnosis compared with the proportion of survivors expected in a set of comparable cancer-free persons. These estimates are based on data from NPCR-funded states that met USCS publication criteria and conducted active case follow-up or linkage with CDC’s National Center for Health Statistics National Death Index (4). For this report, 30 states met these criteria, covering 71% of the U.S. population. The 5-year relative survival rates were calculated for cases diagnosed during 2003–2010 with follow-up through 2010 (4).

In 2011, a total of 1,532,066 invasive cancers were diagnosed and reported to central cancer registries in the United States (excluding Nevada), including 786,102 among males and 745,964 among females (Table 1). The age-adjusted annual incidence for all cancers was 451 per 100,000 population: 508 per 100,000 in males and 410 per 100,000 in females. Among persons aged <20 years, 14,754 cancer cases were diagnosed in 2011 (Table 1). By age group, rates per 100,000 population in 2011 were 18 among persons aged <20 years, 154 among those aged 20–49 years, 816 among those aged 50–64 years, 1,840 among those aged 65–74 years, and 2,223 among those aged ≥75 years (Table 1).

By cancer site, rates were highest for cancers of the prostate (128 per 100,000 men), female breast (122 per 100,000 women), lung and bronchus (61 per 100,000 persons), and colon and rectum (40 per 100,000 persons) (Table 1). These four sites accounted for half of cancers diagnosed in 2011, including 209,292 prostate cancers, 220,097 female breast cancers, 207,339 lung and bronchus cancers, and 135,260 colon and rectum cancers. In 2011, the cervical cancer incidence rate was 7.5 per 100,000 women, representing 12,109 reported cancers.

By state in 2011, all-sites cancer incidence rates ranged from 374 to 509 per 100,000 persons (Figure). State site-specific cancer incidence rates ranged from 79 to 195 per 100,000 men for prostate cancer, 106 to 153 per 100,000 women for female breast cancer, 29 to 93 per 100,000 persons for lung cancer, 33 to 49 per 100,000 persons for colorectal cancer, and 4.5 to 13.7 per 100,000 women for cervical cancer (Figure). *Healthy People 2020* targets were reached in 37 states for incidence of colorectal cancer and in 28 states for incidence of cervical cancer. Compared with the states and DC, cancer incidence rates in Puerto Rico in 2011 were lower for all-sites cancer (339 per 100,000 persons), lung cancer (17 per 100,000 persons), and breast cancer (93 per 100,000 women), but higher for prostate cancer (150 per 100,000 men), colorectal cancer (43 per 100,000 persons), and cervical cancer (13.5 per 100,000 women).

Among persons with cancer diagnosed during 2003–2010, the 5-year relative survival rate was 65% (Table 2). This percentage was similar for males and females. The 5-year relative survival was highest among those diagnosed with cancer before age 45 years (81%) and decreased with increasing age (Table 2). Among the most common cancer sites, 5-year relative survival was highest for prostate cancer (97%) and breast cancer (88%), intermediate for colorectal cancer (63%), and lowest for lung cancer (18%) (Table 2). The 5-year relative survival after any cancer diagnosis was lower for black persons (60%) than for white persons (65%) and for each cancer site (Table 2).

## Discussion

This report provides estimates of cancer incidence for 2011 in the United States and shows that *Healthy People 2020* targets were achieved in 37 states for reduced colorectal cancer incidence and 28 states for reduced cervical cancer incidence. For the first time, cancer incidence rates in Puerto Rico are included with the state-specific cancer incidence rates. Cancer incidence rates in Puerto Rico reflect screening practices and risk factors that might differ from those in the U.S. states.

Also for the first time, data on survival are included. In the United States, about two of three persons diagnosed with cancer survive ≥5 years after diagnosis. This depends on the type of cancer and age at diagnosis, and was lower among black persons compared with white persons. Differences in survival after cancer diagnosis might be attributable to differences in type of cancer, stage at diagnosis, timeliness of follow-up after diagnosis, appropriate treatment after diagnosis, or having a chronic condition (5). Cancer itself is considered a chronic condition, and many cancer survivors face physical, psychological, social, spiritual, and economic challenges because of their cancer diagnosis and treatment (6). CDC strives to address health and quality-of-life issues of cancer survivors through programs and research related to coordination of care, patient-provider communication, health promotion, supportive services, fertility preservation, and health equity.**

Cancer incidence and survival data can guide the planning and evaluation of cancer prevention and control programs. In Vermont, for example, cancer registry data were used to identify two counties with high melanoma incidence rates in which to pilot a new program for skin cancer prevention (7). These data can also assist long-term planning for cancer diagnostic and treatment services. The Colorado Central Cancer Registry, in collaboration with CDC, has built a free, user-friendly web-based module for clinicians that uses cancer registry data to create treatment summaries and personalized cancer survivorship plans (8). Finally, these data can help public health officials set priorities for allocating health resources. For example, data from the North Carolina Central Cancer Registry are linked into North Carolina’s Integrated Cancer Information and Surveillance System, which overlays the cancer data with census data, health indicators, and socioeconomic variables to facilitate cancer-focused research, from prevention through diagnosis, treatment, survival, and end-of-life care (9). CDC annually provides cancer surveillance via several products, including USCS, CDC WONDER, State Cancer Profiles, and CDC’s National Center for Health Statistics Research Data Centers.††

The findings in this report are subject to at least three limitations. First, analyses based on race and ethnicity might be biased if race and ethnicity were systematically misclassified; ongoing efforts are made to ensure that this information is as accurate as possible.§§ Second, delays in cancer reporting might result in an underestimate of certain cancers; reporting delays are more common for cancers such as melanoma that are diagnosed and treated in nonhospital settings such as physicians’ offices (10). Finally, relative survival rates could be calculated only for white and black racial groups because accurate life tables were not available for other racial/ethnic groups.

National cancer surveillance data are essential for public health officials to monitor cancer incidence, mortality, and survival in the United States; identify populations that might benefit most from targeted cancer prevention and control efforts; help guide the planning of health care allocation and support services; and track progress toward the national cancer objectives set forth in *Healthy People 2020*.

What is already known on this topic?

Cancer is a leading cause of illness in the United States. Because of earlier detection of cancers with effective treatments, improved cancer treatments, and better general medical care, the percentage of persons living after a cancer diagnosis has increased over the past decades.

What is added by this report?

National cancer surveillance data indicate that 1,532,066 new cases of invasive cancer were diagnosed in the United States (excluding Nevada) in 2011, an annual incidence rate of 508 cases per 100,000 among males and 410 among females. All-sites cancer incidence rates ranged by state from 374 to 509 per 100,000 persons and was 339 per 100,000 persons in Puerto Rico. *Healthy People 2020* targets were reached in 37 states for reduced incidence of colorectal cancer and in 28 states for reduced incidence of cervical cancer. About two of three persons diagnosed with cancer survived ≥5 years after diagnosis.

What are the implications for public health practice?

Public health officials can use cancer incidence and survival data to identify population groups with high cancer incidence rates and low cancer survival rates who might benefit most from targeted cancer prevention and control efforts. Using these data to effectively develop comprehensive cancer control programs, including supporting the needs of cancer survivors, can help reduce cancer incidence and improve survival.

## Figures and Tables

**FIGURE:**
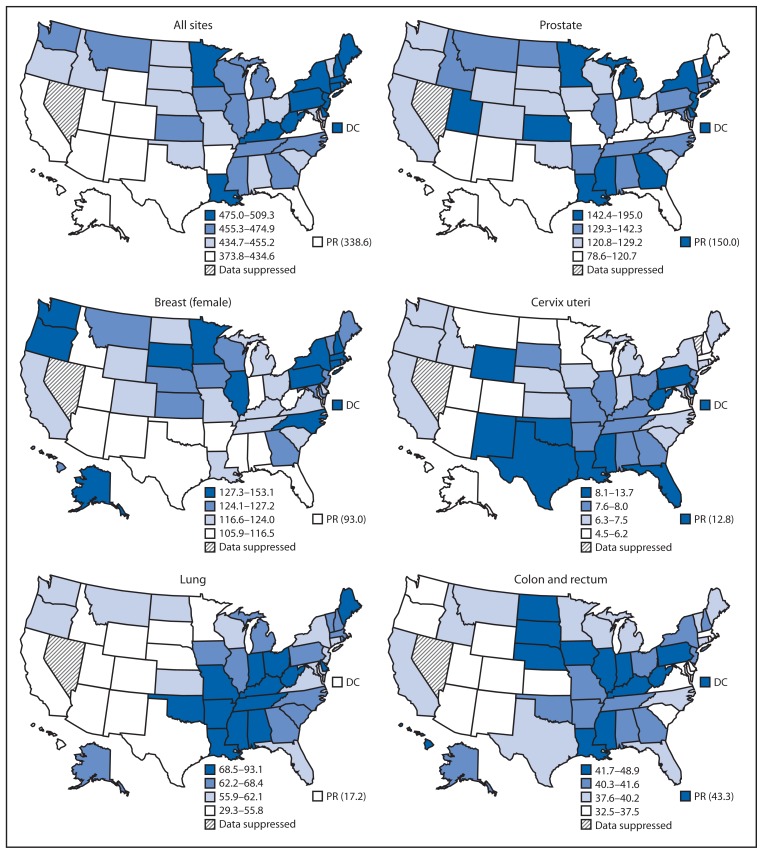
Rate* of invasive cancer, by primary cancer site — National Program of Cancer Registries and Surveillance, Epidemiology, and End Results Program, United States, 2011 * Per 100,000 persons, age-adjusted to the 2000 U.S. standard population.

**TABLE 1:** Number of invasive cancers* and annual rate,† by sex, primary site, race/ethnicity,§ and age group — National Program of Cancer Registries, and Surveillance, Epidemiology, and End Results Program, United States,¶ 2011

	Overall			Males			Females		
			
Characteristic	Rate	No.	(%)	Rate	No.	(%)	Rate	No.	(%)
All sites	450.6	1,532,066		507.5	786,102		410.3	745,964	
Prostate	NA	209,292	(14)	128.3	209,292	(27)	NA	NA	
Female breast	NA	220,097	(14)	NA	NA		122.0	220,097	(30)
Late-stage female breast	NA	73,485		NA	NA		41.4	73,485	
Lung and bronchus	61.0	207,339	(14)	73.0	110,322	(14)	52.0	97,017	(13)
Colon and rectum	39.9	135,260	(9)	46.1	70,099	(9)	34.9	65,161	(9)
Cervix uteri	NA	12,109	(1)	NA	NA		7.5	12,109	(2)
**Race/Ethnicity**									
White	449.7	1,286,265	(84)	499.7	658,861	(84)	414.8	627,404	(84)
Black	458.3	165,062	(11)	554.5	84,664	(11)	393.8	80,398	(11)
American Indian/Alaska Native	273.4	7,877	(1)	293.5	3,776	(<1)	261.0	4,101	(1)
Asian and Pacific Islander	290.4	43,738	(3)	310.1	19,882	(3)	279.8	23,856	(3)
Hispanic	350.6	109,279	(7)	393.5	53,066	(7)	324.2	56,213	(8)
**Age group (yrs)**									
0–19	17.9	14,754	(1)	18.4	7,780	(1)	17.3	6,974	(1)
20–49	154.3	189,430	(12)	114.2	70,352	(9)	194.0	119,078	(16)
50–64	816.1	505,334	(33)	887.1	267,543	(34)	750.6	237,791	(32)
65–74	1,840.0	406,275	(27)	2,258.3	231,725	(29)	1,477.5	174,550	(23)
≥75	2,223.2	416,273	(27)	2,819.2	208,702	(27)	1,830.3	207,571	(28)

**Abbreviation:** NA = not available.

* Excludes basal and squamous cell carcinomas of the skin except when these occur on the skin of the genital organs, and *in situ* cancers except urinary bladder.

† Per 100,000 persons, age-adjusted to the 2000 U.S. standard population.

§ Racial categories are not mutually exclusive from Hispanic ethnicity. Rates are not presented for persons with unknown or other race.

¶ Compiled from cancer registries that meet the data quality criteria for all invasive cancer sites combined (covering approximately 99% of the U.S. population).

**TABLE 2:** 5-year relative survival (percentage) after cancer diagnosis,* by race, sex, primary site, and age group — National Program of Cancer Registries, United States†

	All races			White			Black		
			
Characteristic	Overall	Males	Females	Overall	Males	Females	Overall	Males	Females
**All sites**	**65**	**65**	**65**	**65**	**65**	**66**	**60**	**62**	**57**
Prostate	**NA**	97	NA	NA	97	NA	NA	96	NA
Female breast	**NA**	NA	88	NA	NA	89	NA	NA	79
Lung and bronchus	**18**	15	21	18	16	21	15	13	18
Colon and rectum	**63**	63	64	64	63	64	57	56	59
Cervix uteri	**NA**	NA	68	NA	NA	69	NA	NA	58
**Age group at diagnosis (yrs)**									
0–44	**81**	76	84	82	77	85	70	63	74
45–54	**71**	66	76	73	66	78	62	60	65
55–64	**68**	68	69	69	68	70	63	65	59
65–74	**64**	67	60	64	66	61	60	66	52
≥75	**52**	55	49	52	55	50	45	50	40

**Abbreviation:** NA = not available.

* Based on cases diagnosed during 2003–2010 and follow-up of patients through 2010.

† Compiled from 30 cancer registries that met data quality criteria for survival analysis, covering approximately 71% of the U.S. population.

## Footnotes

* As of 2014, *Healthy People 2020* objectives included improving to 71.7% the proportion of persons surviving ≥5 years after cancer diagnosis, reducing colorectal cancer incidence to 41.6 per 100,000 persons, reducing late-stage breast cancer incidence to 38.9 per 100,000 women, and reducing cervical cancer incidence to 7.5 per 100,000 women.

† Cancer registries demonstrated that cancer incidence data were of high quality by meeting the six USCS publication criteria: 1) case ascertainment is ≥90% complete; 2) ≤5% of cases are ascertained solely on the basis of a death certificate; 3) ≤3% of cases are missing information on sex; 4) ≤3% of cases are missing information on age; 5) ≤5% of cases are missing information on race; and 6) ≥97% of the registry’s records passed a set of single-field and inter-field computerized edits that test the validity and logic of data components. Additional information available at http://www.cdc.gov/uscs.

§ Additional information available at http://seer.cancer.gov/tools/ssm.

¶ Population estimates incorporate bridged single-race estimates derived from the original multiple race categories in the 2010 U.S. Census. Additional information available at http://seer.cancer.gov/popdata/index.html and http://www.census.gov/popest/topics/methodology.

** Additional information available at http://www.cdc.gov/cancer/survivorship.

†† Additional information available at http://www.cdc.gov/cancer/npcr/datarelease.htm, http://wonder.cdc.gov, http://www.statecancerprofiles.cancer.gov/incidencerates/index.php, and http://www.cdc.gov/rdc/b1datatype/dt131.htm.

§§ Additional information available at http://www.cdc.gov/cancer/npcr/uscs/technical_notes/interpreting/race.htm.
